# Elevated Serum Iron Is a Potent Biomarker for Spirometric Resistance to Cigarette Smoke among Japanese Males: The Takahata Study

**DOI:** 10.1371/journal.pone.0074020

**Published:** 2013-09-09

**Authors:** Yoko Shibata, Sumito Inoue, Akira Igarashi, Keiko Yamauchi, Shuichi Abe, Yasuko Aida, Keiko Nunomiya, Masamichi Sato, Hiroshi Nakano, Kento Sato, Tetsu Watanabe, Tuneo Konta, Yoshiyuki Ueno, Takeo Kato, Takamasa Kayama, Isao Kubota

**Affiliations:** 1 Department of Cardiology, Pulmonology and Nephrology, School of Medicine, Yamagata University, Yamagata City, Yamagata, Japan; 2 Global Center of Excellence Program Study Group, School of Medicine, Yamagata University, Yamagata City, Yamagata, Japan; University of Rochester Medical Center, United States of America

## Abstract

Chronic obstructive pulmonary disease is a common disability among elderly subjects with a heavy cigarette smoking habit. In contrast to the population that is susceptible to smoking, in whom pulmonary function worsens with the length of exposure to cigarette smoke, there are elderly individuals whose pulmonary function is not impaired. However, to date, the characteristics of this resistant smoking population have not been investigated. We aimed to identify a biomarker in individuals in whom lung health is maintained despite smoking. Blood sampling and spirometry were performed on 3,257 subjects who participated in a community-based annual health check in Takahata, Japan, from 2004 to 2006. We selected 117 elderly smokers (age ≥70, Brinkman index ≥600, smoking years ≥30). The ‘smoking resistant’ group met the following criteria: FEV_1_/FVC ≥0.7, and FEV_1%_predicted ≥80. Spirometry was re-evaluated in 147 male, current smokers in 2009. Baseline serum iron (sFe) levels were higher in the smoke resistant group compared with the non-resistant group. In those with low sFe levels, FEV_1_/FVC was reduced in male subjects. These spirometric measures were positively associated with sFe levels in men. Multiple linear regression analysis revealed that sFe levels were predictive for spirometric values, independent of other clinical factors. In addition, sFe levels were predictive for a decline in FEV_1_.Serum iron levels may be a biomarker for the spirometric susceptibility of individuals to cigarette smoke.

## Introduction

Long term cigarette smoking results in impaired pulmonary function, such as chronic obstructive pulmonary disease (COPD) in the elderly population [1]. Cigarette smoke contains many toxic and noxious substances that cause inflammation of the respiratory tract and alveolar walls. Identification of biomarkers in smoking-susceptible individuals is of importance to researchers in pulmonary medicine as it may lead to avoidance of the development of smoking-induced pulmonary disorders, such as COPD. Many genome wide association studies (GWAS) have been performed to identify a “smoking susceptibility” gene [2]–[8]. These studies have provided important information that may be relevant to the pathogenesis of COPD. However, the odds ratio of having the risk alleles for the disease is not high; thus the utility of information about the presence of these genomic alleles in individuals is still questionable [9].

We previously reported that circulating homocysteine levels predicted a decline in FEV_1_ in male continuing smokers participating in an annual health check [10]. We also demonstrated that serum uric acid levels were inversely associated with spirometric measures in a healthy Japanese population [11]. In addition, we showed a relationship between abdominal circumference and retrospective decline in FEV_1_ [12]. This evidence suggested that circulating biomarkers and clinical characteristics may potentially be used to identify a smoking susceptible population.

We previously demonstrated that 35% of male smokers and 40% of female smokers aged ≥70 years have air flow limitation [13]. In contrast to the smoking susceptible population in whom pulmonary function worsens with the length of exposure to cigarette smoke, there are elderly individuals whose pulmonary function is not impaired. However, to date, the characteristics of this smoking resistant population have not been investigated. In this study, we sought to identify biomarker(s) that were associated with resistance to cigarette smoke-induced impairment of pulmonary function, by comparing data for the smoking resistant and non-resistant populations of elderly persons who participated in the annual health check in Takahata, Japan. In this paper, we report a novel association between pulmonary function and serum iron (sFe) levels in male subjects, and that elevated sFe levels are a potent biomarker for spirometric resistance to cigarette smoke.

## Methods

### Study Population

This study formed part of the Molecular Epidemiological Study utilizing the Regional Characteristics of 21^st^ Century Centers of Excellence (COE) Program and the Global COE Program in Japan [13]. The study was approved by the ethics committee of Yamagata University School of Medicine and all participants gave written informed consent.

The study was based on an annual community health check, in which all residents of Takahata town in northern Japan, aged 40 years or older, were invited to participate. From 2004 to 2006, 1,579 males and 1,941 females (a total of 3,520 subjects) were enrolled in the study [10]. Two hundred and sixty-three subjects were excluded from the analysis due to spirometry data that did not meet the specified criteria. The data for a total of 3,257 subjects (1,502 males, 1,755 females) was entered into the final statistical analysis. Subjects used a self-report questionnaire to document their medical histories, smoking habits, current use of medications and clinical symptoms. Eleven males and 10 females were receiving therapy for pulmonary disease. However, information on the precise diagnosis and prescriptions was not available on the database. Eleven males and 18 females were receiving treatment for anaemia. However, information on therapy, such as iron supplementation, was not available. None of the subjects received simultaneous respiratory and anti-anaemia therapy. The lifetime consumption of cigarette smoke was assessed using the Brinkman index (daily number of cigarettes x years). Of 523 male current smokers in visit 1, 32 subjects quit smoking, 10 declined spirometric examination, and 334 did not attend their annual health check in 2009. All continuously smoking subjects (n = 147) who received a second spirometric assessment in 2009 were enrolled in the follow-up study (visit 2).

### Definition of the Cigarette Smoke Resistant Population

Of the 3,257 subjects, 119 elderly smokers were selected according to the following criteria: age ≥70 years; Brinkman index ≥600 cigarette×years; and duration of smoking ≥30 years. Among this population of elderly smokers, those who satisfied the following criteria were defined as the ‘smoking resistant’ group: FEV_1_/FVC ≥0.7 and FEV_1%_predicted ≥80. The remaining elderly smokers (those with FEV_1_/FVC <0.7 or FEV_1%_predicted <80) were defined as the ‘smoking non-resistant’ group. Because the number of female smokers was too small to perform statistical analysis (1 in the resistant group, 1 in the non-resistant group), we excluded the female subjects from the analyses. A total of 117 subjects were included in the final analyses. Among these 117 subjects, six received a second spirometric assessment at Visit 2.

### Measurements

Blood samples were taken from the antecubital vein of subjects who had been fasting, and the samples were immediately transferred to chilled tubes. Serum Fe levels were measured in 3,248 of the 3,257 subjects. Total plasma homocysteine concentrations were measured using an enzymatic homocysteine assay kit (MBL, Nagoya, Japan). Anti-nuclear antibody levels were determined using an enzyme immunoassay method (BML, Tokyo, Japan). Plasma renin activity was determined using a radioimmunoassay (Renin-RIA bead; Abbot Japan, Tokyo, Japan). Spirometric parameters [forced vital capacity (FVC) and forced expiratory volume in 1 s (FEV_1_)] were measured using standard techniques, with subjects performing FVC manoeuvres on a CHESTAC-25 part II EX instrument (Chest Corp., Tokyo, Japan), according to the guidelines of the Japanese Respiratory Society (JRS) [14]. Bronchodilator was not administered prior to spirometry. The highest value from at least three FVC manoeuvres by each subject was used for the analysis. The results were assessed by two pulmonary physicians who visually inspected the flow-volume curves, and excluded subjects with inadequate data, as defined by the JRS criteria [14]. The rate of decline in spirometric measures [ΔFEV_1_/year (%) and ΔFVC/year (%)] were calculated as [(value at second spirometry – value at first spirometry)/value at first spirometry]×100/time between observations (years). Declines in FVC and FEV_1_ were defined as annual rates of decline equivalent to, or greater than, the first quintiles of ΔFVC/year (%) and ΔFEV_1_/year (%), respectively [10].

### Statistical Analysis

For continuous variables, data are presented as mean values (SD). Student’s t test for parametric data and the Mann-Whitney U test for non-parametric data were used to analyse differences between the two groups. For multiple comparisons, analysis of variance (ANOVA) followed by Tukey’s test was performed. Correlations between two variables were evaluated using Pearson’s product moment correlation coefficient. Analysis of covariance (ANCOVA) was performed to determine the difference of the linear regression slopes between two groups. Multiple linear regression analysis was performed to determine whether serum iron levels were associated with spirometric measures after adjustment for all other variables included in the model. The results of multiple logistic regression analyses are presented as odds ratios (OR) with 95% confidence intervals (CI). Statistical significance was inferred for two-sided *P* values <0.05. All statistical analyses were performed using JMP version 8 software (SAS Institute Inc., Cary, NC, USA).

## Results

### Characteristics of the Cigarette Smoke Resistant Group

The differences in pulmonary function of elderly smokers between the ‘cigarette smoke non-resistant’ group and the ‘resistant’ group are summarized in Table 1. FVC, FEV_1_, and FEV_1_/FVC were significantly higher in the resistant group than in the non-resistant group. The differences in the characteristics of the elderly male smokers according to cigarette smoke resistance were assessed (Table 2). Serum Fe concentrations and body mass index were significantly higher in the resistant group than in the non-resistant group, whereas all other factors examined did not differ between the two groups (Table 2).

**Table 1:** Spirometric parameters in elderly male smokers, both resistant and non-resistant to cigarette smoke.

	Cigarette smoke non-resistant n = 57	Cigarette smoke resistant n = 60	*P*
FVC %predicted	88.3 (20.4)	99.4 (13.8)	0.0007
FEV_1%_predicted	73.7 (20.5)	100.2 (13.2)	<0.0001
FEV_1_/FVC	64.4 (11.5)	78.2 (6.2)	<0.0001

The ‘cigarette smoke resistant’ population is defined in *Methods*.

FVC, forced vital capacity; FEV_1_, forced expiratory volume in 1 s.

**Table 2:** Demographic characteristics and laboratory measurements in elderly male smokers, both resistant and non-resistant to cigarette smoke.

	Cigarette smoke non-resistant n = 57	Cigarette smoke resistant n = 60	*P*
Age, years	74.9 (3.4)	74.3 (2.9)	0.356
Body mass index, kg/m^2^	22.3 (3.1)	23.5 (3.3)	0.046
Brinkman index, cigarette years	985.3 (401.6)	973.3 (408.2)	0.873
Red blood cell count, 10^4^/µL	438.8 (48.0)	449.3 (47.3)	0.237
Haemoglobin, g/dL	14.1 (1.5)	14.5 (1.3)	0.208
Haematocrit, %	42.0 (4.5)	42.8 (3.8)	0.291
Serum Fe, µg/dL	107.8 (47.7)	121.6 (38.7)	0.016
Albumin, g/dL	4.4 (0.4)	4.5 (0.3)	0.085
Aspartate aminotransferase, IU/L	26.8 (10.3)	27.7 (9.7)	0.615
Alanine aminotransferase, IU/L	21.5 (13.3)	23.7 (15.3)	0.411
Blood urea nitrogen, mg/dL	16.4 (4.6)	16.1 (3.8)	0.740
Serum creatinine, mg/dL	0.8 (0.2)	0.8 (0.2)	0.424
Uric acid, mg/dL	5.7 (1.3)	5.7 (1.1)	0.916
Haemoglobin A1c, %	5.4 (0.6)	5.3 (0.6)	0.759
Total cholesterol, mg/dL	183.0 (33.3)	184.8 (29.4)	0.757
Triglyceride, mg/dL	99.4 (58.6)	104.6 (41.6)	0.585
High density lipoprotein-cholesterol, mg/dL	54.5 (13.0)	55.8 (13.1)	0.593
Low density lipoprotein-cholesterol, mg/dL	112.9 (31.9)	115.2 (27.0)	0.676
Adiponectin, µg/mL	9.8 (5.7)	9.4 (5.8)	0.725
B-type natriuretic peptide, pg/mL	73.6 (98.9)	37.5 (27.9)	0.366
Plasma renin activity, ng/[mL h]	2.9 (6.0)	2.4 (3.3)	0.580
Angiotensin converting enzyme, U/L	15.6 (7.3)	15.7 (5.9)	0.929
Homocysteine, µM	14.9 (6.6)	13.8 (3.7)	0.249
D-Dimer, µg/mL	0.9 (0.7)	0.7 (0.3)	0.052
Fibrinogen, mg/dL	359.0 (92.9)	357.7 (69.1)	0.932
Serum amylase, U/L	119.2 (43.1)	124.9 (35.5)	0.436
Anti-nuclear antibody index	15.0 (9.3)	14.7 (8.0)	0.845

Among the 117 elderly smokers, homocysteine data was not available for one subject. One subject in the non-resistant group and no subjects in the resistant group were receiving anti-anaemia therapy (chi-square test: *P* = 0.246). One subject in the non-resistant group and two subjects in the resistant group were receiving respiratory medications (chi-square test: *P* = 0.585). Information on diagnoses and medications was not available. Data are mean values (SD). The ‘cigarette smoke resistant’ population is defined in *Methods.*

### Relationship between Serum Iron Levels and Spirometric Measures among the Takahata Study Population Who Performed Spirometry from 2004 through 2006: Cross-sectional Analyses

**Figure 1:**
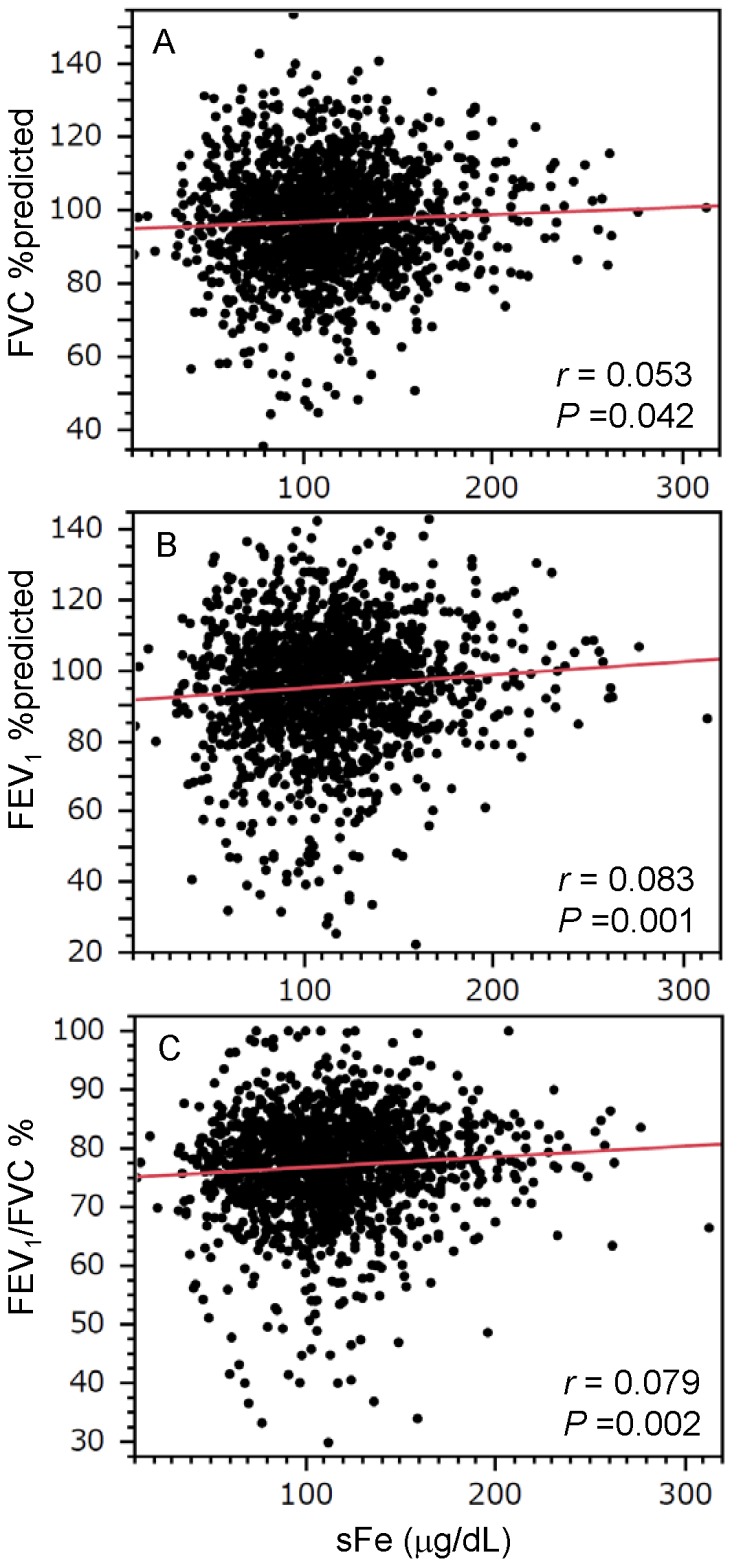
Correlations between spirometric measurements and serum iron levels in male subjects. Graphs show the relationships between spirometric parameters (A: FVC %predicted; B: FEV_1%_predicted; C: FEV_1_/FVC) and serum iron (sFe) levels. Correlations between spirometric measurements and sFe values were evaluated using Pearson’s product moment correlation coefficient. There were positive relationships between these spirometric parameters and sFe levels in males. FVC, forced vital capacity; FEV_1_, forced expiratory volume in 1 s.

In contrast to high percentage of male smokers, that of female smokers were low in this population (male, n = 999, 66.5%; female, n = 161, 9.1%). Due to this difference in smoking behaviour between men and women, we thought that there were not sufficient smoking females in this study population to obtain statistically valid results, and decided to analyse only male data. The mean sFe concentration was 113.6 (38.9) µg/dL (n = 1489). Among males attending Yamagata University Hospital the lower limit of the normal range for sFe was 54 µg/dL. Table 3 shows a comparison of the characteristics and spirometric measures between subjects with a sFe level equal to or greater than the lower limit of the normal range (normal sFe group) and those with a lower sFe level (low sFe group). The mean haemoglobin concentration was significantly lower in the low sFe group than in the normal sFe group. Age, BMI, the percentage of smokers, Brinkman index (overall and current/past smokers), uric acid and homocysteine levels, and FVC and FEV_1_ did not differ between the two groups. However, FEV_1_/FVC was significantly reduced in the low sFe group compared with the normal sFe group (Table 3). As shown in Figure 1, there were positive relationships between sFe levels and spirometric parameters in male subjects. These positive relationships were replicated among the male smokers in this study population (Table 4). In non-smokers, there were statistical significance in relationship between FEV_1_/FVC and sFe level. There was a trend towards a lower FEV_1_ in the low sFe, but this relationship did not reach statistical significance. Correlation of FVC with sFe was not significant (Table 4).The slopes of the linear regression between sFe and spirometric parameters in smokers did not significantly differ from those in non-smokers (ANCOVA: FVC %predicted, P = 0.335; FEV_1%_predicted, P = 0.681; FEV_1_/FVC, P = 0.655). Multivariate linear regression was performed to determine if serum iron levels were cross-sectionally associated with spirometric parameters, independently of age, Brinkman index, haemoglobin concentration, and other factors that previously were reported to be significant in the Takahata Study [10], [11]. As shown in Table 5, 6 and 7, sFe levels were predictive for FVC, FEV_1_ and FEV_1_/FVC in male subjects, independently of age, Brinkman index, and homocysteine, uric acid and haemoglobin levels. When sFe/haemoglobin ratio was used instead of absolute sFe and haemoglobin values, sFe/haemoglobin ratio was also significantly predictive for FVC, FEV_1_ and FEV_1_/FVC in male subjects, independently of age, Brinkman index, and homocysteine and uric acid levels (FVC, *P* = 0.0418; FEV_1_, *P* = 0.0004; FEV_1_/FVC, *P* = 0.0028).

**Table 3:** Differences in characteristics and spirometric measures according to serum Fe level in male subjects.

	Fe <54 µg/dL n = 60	Fe ≥54 µg/dL n = 1440	*P*
age, years	64.1 (10.0)	62.8 (10.4)	0.354
BMI, kg/m^2^	23.1 (3.0)	23.5 (3.0)	0.360
percentage of current and past smokers, %	56.7	66.9	0.105
Brinkman index overall, cigarette×year	365.2 (474.8)	444.5 (496.4)	0.273
Brinkman index in current and past smokers, cigarette×year	778.0 (395.1)	738.7 (438.3)	0.672
haemoglobin, g/dL	13.6 (1.9)	14.7 (1.2)	<0.0001
uric acid, mg/dL	5.6 (1.4)	5.8 (1.3)	0.192
homocysteine, µM	11.6 (3.5)	12.6 (7.0)	0.254
FVC %predicted	97.5 (13.9)	97.3 (14.9)	0.911
FEV_1%_predicted	93.3 (17.7)	95.7 (17.5)	0.312
FEV_1_/FVC, %	74.7 (8.0)	77.1 (8.9)	0.038

Data are mean values (SD). Data for sFe, haemoglobin, uric acid and homocysteine levels, and for Brinkman index were not available for 2, 2, 2, 29 and 266 of the 1502 male subjects, respectively. Eight subjects in the normal sFe group and three subjects in the low sFe group were receiving anti-anaemia therapy (chi-square test: *P* = 0.266). Nine subjects in the normal sFe group and two subjects in the low sFe group were receiving respiratory therapy (chi-square test: *P* = 0.729). Information on diagnoses and medications was not available.

BMI, body mass index; FVC, forced vital capacity; FEV_1_, forced expiratory volume in 1 s.

**Table 4:** Correlations between serum iron levels and spirometric parameters in male non-smokers and current/past smokers.

	Non-smokers		Current and past smokers	
	*r*	*P*	*r*	*P*
FVC %predicted	0.022	0.614	0.076	0.016
FEV1%predicted	0.084	0.059	0.102	0.001
FEV1/FVC	0.117	0.009	0.081	0.011

FVC, forced vital capacity; FEV_1_, forced expiratory volume in 1 s.

**Table 5:** Multivariate linear regression analysis for factors predictive for forced vital capacity (FVC) % predicted in male subjects.

	Coefficient	SE	*P*
Age	−0.055	0.044	0.205
Brinkman index	−0.005	0.001	<0.0001
sFe	0.023	0.011	0.038
homocysteine	−0.059	0.061	0.329
uric acid	−0.599	0.332	0.072
Haemoglobin	0.074	0.368	0.839

Variables that were possibly associated with FVC % predicted, including Brinkman index, and homocysteine and uric acid levels were selected. To adjust for age, this parameter was also included in the analyses. Since sFe levels are strongly associated with haemoglobin concentration, the latter was also included as a variable. Data for sFe, haemoglobin, uric acid and homocysteine levels, and for Brinkman index was not available for 2, 2, 2, 29 and 266 of the 1502 male subjects, respectively. Data for a total of 1212 subjects was included in the final analyses.

**Table 6:** Multivariate linear regression analysis for factors predictive for forced expiratory volume in 1(FEV_1_) % predicted in male subjects.

	Coefficient	SE	*P*
Age	−0.181	0.05	0.0003
Brinkman index	−0.009	0.001	<0.0001
sFe	0.045	0.013	0.0005
homocysteine	−0.052	0.069	0.456
uric acid	−0.652	0.379	0.085
Haemoglobin	−0.2	0.42	0.634

Variables that were possibly associated with FEV_1%_ predicted, including Brinkman index, and homocysteine and uric acid levels were selected. To adjust for age, this parameter was also included in the analyses. Other details are described in the legend of Table 5.

**Table 7:** Multivariate linear regression analysis for factors predictive for forced expiratory volume in 1s (FEV_1_)/forced vital capacity (FVC) in male subjects.

	Coefficient	SE	*P*
Age	−0.209	0.025	<0.0001
Brinkman index	−0.004	0.000	<0.0001
sFe	0.018	0.006	0.004
homocysteine	−0.002	0.034	0.943
uric acid	−0.135	0.187	0.47
Haemoglobin	−0.178	0.208	0.389

Variables that were possibly associated with FEV_1_/FVC, including Brinkman index, and homocysteine and uric acid levels were selected. To adjust for age, this parameter was also included in the analyses. Other details are described in the legend of Table 5.

### Serum Iron Levels and Decline in Spirometric Parameters: Longitudinal Analyses

Logistic regression analyses were performed to determine if serum iron levels were associated with a decline in spirometric parameters between the first (2004–2006) and second visits (2009) [10]. The characteristics of the male subjects who maintained their smoking habit from visit 1 through visit 2 (Group 1) are summarized in Table 8. BMI, Brinkman index, and the levels of haemoglobin, sFe, homocysteine and uric acid in these 147 subjects (Group1 in Table 8) did not differ significantly from those of male smokers who did not receive spirometric assessment at Visit 2 (Groups 2, 3 and 4 in Table 8). These 147 continuing smokers were significantly younger than those who did not attend an annual health check in 2009 (Group 4 in Table 8), and also had higher FEV_1_/FVC ratios than subjects in Group 4. At Visit 2, FVC %predicted increased significantly and FEV_1_/FVC decreased significantly compared with the values at Visit 1, whereas FEV_1%_predicted remained unchanged (Table 8). In these subjects, only five and one were classified as elderly resistant and non-resistant smokers, respectively. Univariate analyses demonstrated that sFe level was predictive for a decline in FEV_1_ (Table 10), but not for a decline in FVC (Table 9). In addition, multivariate analysis demonstrated that sFe levels were predictive for a decline in FEV_1_, independent of age, Brinkman index, and homocysteine, uric acid and haemoglobin levels (Table 10). When sFe/haemoglobin ratio was used instead of absolute sFe and haemoglobin values, sFe/haemoglobin ratio was also significantly predictive for a decline in FEV_1_ in male subjects, independently of age, Brinkman index, and homocysteine and uric acid levels [multivariate logistic regression analysis: sFe/haemoglobin (per 1 SD increase) OR 0.546, 95% confidence interval 0.307–0.908; *P* = 0.0185].

**Table 8:** Characteristics of the male smokers at Visit 1 compared with those of subjects receiving spirometric assessment at Visit 2.

	Group 1 (n = 147)	Group 2 (n = 32)	Group 3 (n = 10)	Group 4 (n = 334)	*P* (ANOVA)
age, years	56.4 (9.1)	60.1 (7.7)	61.3 (6.5)	61.2 (10.9)*	<0.0001
Body mass index, kg/m2	22.8 (2.8)	23.3 (2.0)	24.1 (2.6)	22.9 (3.1)	0.454
Brinkman index, cigarette·year	751.7 (413.8)	661.7 (293.6)	859.4 (356.1)	762.3 (343.9)	0.439
haemoglobin, g/dL	15.1 (1.2)	14.8 (0.8)	14.8 (1.1)	14.8 (1.2)	0.243
sFe, µg/dL	120.2 (45.2)	116.1 (36.7)	111.5 (51.8)	117.2 (41.0)	0.273
homocysteine, µM	12.4 (4.4)	11.7 (2.4)	15.1 (4.7)	13.8 (12.1)	0.403
uric acid, mg/dL	5.8 (1.2)	5.7 (1.2)	5.5 (0.6)	5.6 (1.3)	0.729
FVC %predicted _visit 1_	94.4 (13.5)	99.1 (13.2)	98.4 (13.2)	94.7 (15.5)	0.342
FEV_1%_predicted _visit 1_	92.4 (15.9)	97.6 (13.2)	91.6 (12.4)	90.4 (18.6)	0.136
FEV_1_/FVC_ visit 1_, %	77.3 (8.4)	77.9 (7.8)	73.6 (7.9)	74.8 (10.1)*	0.031
FVC %predicted _visit 2_	98.5 (14.4)#	–	–	–	–
FEV_1%_predicted_ visit 2_	91.9 (17.1)	–	–	–	–
FEV_1_/FVC_ visit 2_, %	73.0 (9.3)##	–	–	–	–

Group 1: continuing smokers who received spirometric assessment at Visit 2; Group 2: those who quit smoking before Visit 2; Group 3: continuing smokers who refused spirometric assessment at Visit 2; Group 4: subjects who did not participate in the annual health check in 2009.

One subject in Group 1 was receiving anti-anaemia therapy. No subjects were receiving respiratory therapy.

* *P*<0.05 vs Group 1 by ANOVA followed by Tukey’s test.

# *P*<0.05,

## *P*<0.001 vs Visit 1 by Student’s t-test.

Data are mean values (SD). Data for homocysteine levels was not available for four male subjects.

BMI, body mass index; FVC, forced vital capacity; FEV_1_, forced expiratory volume in 1 s; SE, standard error; sFe, serum iron.

**Table 9:** Univariate logistic regression analyses for factors that were predictive for decline in forced vital capacity from first assessment (2004–2006) through second assessment (2009).

Univariate	OR	95% CI	*P*
sFe (per 1 SD increase)	0.756	(0.475, 1.158)	0.204

CI, confidence interval; OR, odds ratio; SD, standard deviation; sFe, serum iron.

**Table 10:** Univariate and multivariate logistic regression analyses for factors that were predictive for decline in forced expiratory volume in 1(2004–2006) through second assessment (2009).

Univariate	OR	95% CI	*P*
sFe (per 1 SD increase)	0.576	(0.348, 0.906)	0.016
Multivariate	OR	95% CI	*P*
age (per 1 SD increase)	1.445	(0.860, 2.490)	0.170
Brinkman index (per 1 SD increase)	1.451	(0.850, 2.448)	0.157
sFe (per 1 SD increase)	0.535	(0.297, 0.898)	0.026
homocysteine (per 1 SD increase)	1.669	(1.086, 2.598)	0.017
uric acid (per 1 SD increase)	0.802	(0.494, 1.279)	0.360
haemoglobin(per 1 SD increase)	1.118	(0.641, 1.966)	0.693

CI, confidence interval; OR, odds ratio; SD, standard deviation; sFe, serum iron.

## Discussion

In this study, elderly subjects who had long-term exposure to cigarette smoke were selected from the total study population, and were divided into two groups: ‘cigarette smoke resistant’ and ‘non-resistant’, according to whether their spirometry was normal or abnormal. Elevated serum iron levels were demonstrated in the cigarette smoke resistant group compared with the non-resistant group. Among subjects in the Takahata Study who performed spirometry in 2004–2006, male FEV_1_/FVC was reduced in the group with low sFe levels. There were positive associations between those spirometric measures and sFe levels in men. In addition, sFe levels were predictive for a decline in FEV_1_, independent of Brinkman index, and homocysteine and uric acid levels.

To determine whether sFe level was correlated with pulmonary function only smokers or in all individuals, we assessed these relationships in non-smokers and smokers, separately. As shown in Table 4, there was significant correlation between sFe and male FEV_1_/FVC even in non-smokers. In contrast to non-smokers, there were clear relationships between sFe and pulmonary functions, FVC, FEV_1_ and FEV_1_/FVC, in smokers. However, ANCOVA failed to demonstrate the difference of the linear regression slope between smokers and non-smokers, suggesting that cigarette smoking did not enhance the relationship between sFe and pulmonary functions. It seems that sFe level is correlated with pulmonary function in male individuals including non-smokers.

Inhalation of cigarette smoke increases carbon monoxide levels, leading to increases in haemoglobin levels in smokers [15]. Levels of sFe were significantly associated with blood haemoglobin concentrations. Therefore, to elucidate the possibility that the true factor involved in the increased tolerance to carbon monoxide is an association with spirometric resistance to cigarette smoking, we performed multiple regression analyses with haemoglobin concentration or sFe/haemoglobin ratio as one of the covariates (Tables 5, 6, 7 and 10). In both these analyses, sFe adjusted for possible confounding factors, including haemoglobin levels and sFe/haemoglobin ratio, was significantly predictive for pulmonary function and decline in FEV_1_. These results suggest that the association between pulmonary function and sFe is independent of factors involved in tolerance to increased carbon monoxide levels.

It is estimated that 50% of heavy smokers develop COPD [16]. Many studies in this research area have focused on the population of patients with COPD or subjects with reduced pulmonary function. To the best of our knowledge, no study focusing on the spirometric resistance of subjects to cigarette smoke has been reported. From this point of view, our study was unique and valuable because a previously unknown relationship between serum iron levels and pulmonary function in the general population was identified. To date, very few studies have investigated this relationship. McKeever et al. demonstrated that higher serum levels of iron were associated with higher FEV_1_ values [17]. However, the mechanism underlying this phenomenon remains unclear.

Cigarette smoking is a major cause of lung disorders such as COPD and lung cancer. Because cigarette smoke consists of thousands of oxidants, long-term exposure induces oxidative stress, not only in the lung, but also systemically. As the main cause of decline in FEV_1_ and airflow limitation in this elderly population is thought to be long term exposure to cigarette smoke [13], the effects of cigarette smoking on iron metabolism need to be discussed. Ghio et al. demonstrated that inhalation of cigarette smoke alters iron homeostasis [18]. In rats exposed to cigarette smoke, iron levels in bronchoalveolar lavage fluid were increased; whereas serum iron levels were decreased. Furthermore, the expression of divalent metal transporter 1 was significantly increased in the lungs of smoke exposed rats. They also showed that iron levels in bronchoalveolar lavage of smokers without COPD and patients with COPD were significantly higher than those in non-smokers, and that the levels in patients with COPD were significantly higher than those in smokers without COPD [18]. The findings from the study by Ghio et al. suggest that inhalation of cigarette smoke results in the transport of serum iron to the lung, and the consequent reduction of serum iron levels. Since iron is an important source of Fenton reactions [19], the accumulation of iron may cause lung inflammation through superoxide generation, leading to impairment of the respiratory system. Future analyses of iron concentrations in smokers and patients with COPD may aid further understanding of the association between pulmonary function and sFe levels.

The induction of haem oxygenase-1 (HO-1) reportedly results in elevated serum iron levels in mice [20]. HO-1 plays important roles as an antioxidant in the lung [21], and is thought to be associated with the pathogenesis of COPD [21]. The prevalence of COPD was reported to be higher in subjects who carry the long (GT)_n_ repeat in the promoter region of the HO-1 gene compared with those who carry the smaller (GT)_n_ repeat [22]. Furthermore, the presence of the longer (GT)_n_ repeat was demonstrated to reduce the activity of the HO-1 gene promoter [22], and HO-1 expression in alveolar macrophages of patients with severe COPD was reduced compared with that in smokers whose pulmonary function was normal [23]. Therefore, increased expression of HO-1, with its antioxidant properties, may prevent damage to the airways induced by inhalation of cigarette smoke. Based on this evidence, the difference in serum iron levels between subjects with or without resistance to cigarette smoke may reflect the individual capacity to resist oxidative stress through antioxidants such as HO-1. Although information on the levels of HO-1 expression and genotype in the promoter region of HO-1 were not available in this study, individuals whose serum iron levels were high may have had greater protection against oxidative stress compared to those with low serum iron levels, resulting in the preservation of FEV_1_ despite long term inhalation of cigarette smoke.

Lack of genetic information on iron metabolism, and genotypes at the promoter region of HO-1 that regulate the expression of this enzyme, was an important limitation of this study. Most epidemiological studies, including the present study, have similar limitations, and to elucidate the precise mechanism underlying the phenomenon observed in this study more detailed studies are required. Another limitation of the present study was the lack of information on iron intake in the study population. Low intakes of iron have been reported among young smokers in Korea [24]. The difference in serum levels of iron between smoke resistant and non-resistant subjects may be attributable to differences in daily intake of iron from food. In addition, differences between the two groups in type of employment, socio-economic status or life styles may have influenced the results. However, this information was also not available in the present study. Moreover, a little possibility regarding to the false discovery still remained because this study was assessing many variables. Further studies, including the same analysis in another population, are required to address these limitations. In addition, studies are required to analyse the effects of cigarette smoking on iron metabolism, and the association between differences in genotype and iron metabolism in these two groups of subjects.

In conclusion, we demonstrated elevated serum iron levels in an elderly population that was spirometrically resistant to cigarette smoke. Serum iron levels may be a simple biomarker indicating resistance to the lung toxicity of cigarette smoke. The mechanism underlying the relationship between serum iron levels and pulmonary function remains to be determined, and the question still remains whether the elevation of serum iron has a protective effect in the lung. This elevation may simply depend on the degree of the individual response to cigarette smoke stimulation, and the transfer of iron from serum to lung tissues may be less in the smoke resistant group compared with the non-resistant group, resulting in higher serum iron levels in the smoke resistant group. Future studies investigating the longitudinal effects of iron supplementation or iron chelation on the decline in pulmonary function in cigarette smokers may help increase our understanding of this phenomenon.
